# Reduced volume of the mediodorsal and anteroventral thalamus is associated with anxiety in Parkinson's disease: A cross-sectional 7-tesla MRI study

**DOI:** 10.1177/1877718X241308141

**Published:** 2025-02-03

**Authors:** Guillaume Carey, Mark L Kuijf, Stijn Michielse, Amée F Wolters, Kathy Dujardin, Albert FG Leentjens

**Affiliations:** 1 School for Mental Health and Neurosciences (MHeNS), Maastricht University, Maastricht, The Netherlands; 2 Univ. Lille, Inserm, CHU Lille, U1172 - LilNCog - Lille Neuroscience & Cognition, Lille, France; 3 Department of Neurology and Movement Disorders, Lille University Medical Centre, Lille, France; 4 Department of Neurology, Maastricht University Medical Centre, Maastricht, The Netherlands; 5 Department of Neurology, Catharina Hospital Eindhoven, Eindhoven, The Netherlands; 6 Department of Psychiatry, Maastricht University Medical Centre, Maastricht, The Netherlands

**Keywords:** thalamus, mediodorsal, fear, Parkinson, anxiety, magnetic resonance imaging

## Abstract

**Background:**

Parkinson's disease (PD)-related anxiety occurs frequently and may be associated with imbalance between anxiety-related circuits. While the thalamus is a shared region of these circuits, its role in PD-related anxiety has not been explored so far.

**Objective:**

To identify changes in volume of the thalamus and its subnuclei in patients with PD-related anxiety.

**Methods:**

Cognitively intact PD patients (n = 105) were divided into two groups based on their score on the Parkinson anxiety scale (PAS): 31 PD patients had anxiety (Anx-PD) and 74 did not have anxiety (non-Anx-PD). Forty-five healthy control subjects were included. Participants underwent 7-Tesla MRI scanning. Using automatic segmentation, the volumes of the thalamus and its subnuclei were measured, compared between the groups and regressed on the PAS.

**Results:**

The volumes of the thalamus and its subnuclei did not significantly differ between the groups. However, in anxious PD patients, more severe anxiety was strongly associated with a smaller volume of the right medial thalamic subregion, specifically the right mediodorsal magnocellular nucleus and the right mediodorsal parvocellular nucleus (R = 0.63, ß_PAS _= −0.546, p-value_model _= 0.007 and R = 0.60, ß_PAS _= −0.547, p-value_model _= 0.016, respectively), and of the left anteroventral thalamus (R = 0.73, FDR p-value_model _= 0.002, ß_PAS _= −0.407, p-value_PAS _= 0.01).

**Conclusions:**

A reduced volume of the mediodorsal and anteroventral thalamus, overlapping structures between the anxiety related circuits, are associated with more severe PD-related anxiety and may explain its high prevalence in the disease.

## Plain language summary

Anxiety is a frequent and debilitating symptom in Parkinson's disease. Its management and treatment is challenging and a better understanding of the underlying mechanisms may help treating it better. It is hypothesized that the balance of two anxiety circuits in the brain, the so-called fear circuit and the limbic anxiety circuit, is disturbed in Parkinson-related anxiety. There is an overactivation of the fear circuit and an underactivation of the limbic anxiety circuit. These circuits overlap in part and studying alterations of the overlapping structures may help to better understand the mechanisms of Parkinson-related anxiety. The thalamus is an important relay station in the brain, which was shown to be involved in Parkinson-related anxiety. The objective of this study was to identify changes of the volume of the thalamus and its subregions in Parkinson-related anxiety using high-definition MRI scanning. We hypothesized that the volume of some parts of the thalamus would be reduced in Parkinson-related anxiety. We included 105 Parkinson patients, of which 31 had significant anxiety and 74 did not, and 45 healthy participants. All participants underwent a clinical and a psychological assessment and a high-definition “7-Tesla” MRI scan in order to measure the volume of the thalamus and its subregions. The volume of the thalamus was the same in the three groups but a reduced volume of some sub-parts, the medio-dorsal and antero-ventral thalamus, was associated with more severe anxiety in Parkinson patients. These two thalamic parts are part of both the fear circuit and the limbic anxiety circuit. Knowledge of the interrelationship of the two circuits and their imbalance in Parkinson-related anxiety could open new avenues of treatment and management of Parkinson-related anxiety.

## Introduction

Parkinson's disease (PD) is a neurodegenerative disorder characterized by both motor and non-motor symptoms. Anxiety is one of the most common neuropsychiatric symptoms in PD, with an average point prevalence around 31%.^1^ PD-related anxiety is associated with increased motor disability and a reduced quality of life.^2^ While the potential involvement of various anatomical structures in PD-related anxiety was suggested,^3–5^ findings are inconsistent and in need of confirmation. Recent studies suggested that PD-related anxiety could result from an imbalance between the two anxiety-related circuits, i.e., the fear circuit and the limbic anxiety circuit.^6,7^ In PD, striatal dopamine depletion could lead to reduced activity in the limbic anxiety circuit while the fear circuit would become relatively overactivated.^6^

The limbic anxiety circuit, involved in emotional and behavioral adaptations to fear, connects the anterior cingulate cortex, the medial prefrontal cortex (PFC) and brainstem nuclei with the nucleus accumbens, the pallidum, the subthalamic nucleus and the thalamus to modulate mood and behavior.^8^ The fear circuit, involved in fear processing, connects the amygdala to the anterior cingular cortex, the medial PFC, the insular cortex, the hippocampus, the striatum and the thalamus.^9,10^

The overlap between these two circuits mainly concerns the striatum (i.e., nucleus accumbens), brainstem nuclei (i.e., locus coeruleus, ventral tegmental area, substantia nigra) and the thalamus.^6^ In a previous study, we reported that functional connectivity between the orbitofrontal cortex and the thalamus was increased after cognitive behavioral therapy for PD-related anxiety suggesting that thalamic dysfunction could be associated with these symptoms.^11^ So far, the role of the thalamus in the development of anxiety in PD was never explored. High-field imaging and the use of the recent high-field imaging atlas of the thalamic subregions may advance our understanding of its role.^12,13^

The aim of this study was to identify anatomical changes in the volume of the thalamus and the thalamic subnuclei in patients with PD-related anxiety compared to patients without anxiety, using 7-Tesla MRI. We hypothesized that the volume of the thalamic subnuclei could be smaller in patients with PD-related anxiety compared to patients without anxiety and healthy controls, especially those involved in emotion and behavior such as the anteroventral, the laterodorsal, the mediodorsal thalamus and the pulvinar.

## Methods

### Study design

Data came from the TRACK-PD study.^14^ In this on-going longitudinal observational study, non-demented PD patients and healthy controls (HC) are included, with a 4-years follow-up. All data were collected at Maastricht University Medical Centre. A 7-Tesla brain MRI as well as demographical, clinical, cognitive, and neuropsychiatric information were recorded. In the current study, we only focused on the baseline dataset. The study design is published previously and detailed by Wolters et al.^14^ Ethical approval was provided by the local medical ethical committee and the study has been registered (Dutch Trial Register, NL7558). Written informed consent was obtained from all participants.

### Study population

In this study, patients diagnosed with PD in the previous three years were recruited among outpatients of the Movement Disorder Clinic of Maastricht University Medical Centre and collaborating hospitals. In addition, other media, such as websites, social media, and patient meetings were used to recruit patients. HC participants were recruited through advertisements in the hospital and university. Participants included in this study were diagnosed with PD by a neurologist based on the Movement Disorder Society (MDS) clinical diagnostic criteria,^15^ had a score equal or higher than 24 on the Montreal Cognitive Assessment (MoCA) at baseline,^16^ were able to read and understand Dutch, were 18 years of age or older and provided written informed consent. Participants with advanced cognitive impairment, defined as a score of <24 on the MoCA, or a diagnosis of dementia according to the criteria of the fifth edition of the Diagnostic and Statistical Manual of Mental Disorders (DSM 5), at baseline, or diagnosed with a neurodegenerative disease other than PD, or with any contra-indications for a 7-T MRI scan (such as claustrophobia, permanent make-up or the presence of incompatible metallic devices in their body) were excluded. These exclusion criteria were also in place for the HC group.

Demographic and clinical variables were recorded including age, sex, handedness, disease duration, and the total levodopa equivalent daily dose (LEDD). Motor symptoms and disease severity were respectively assessed during “ON'’ phases with the Movement Disorder Society – unified Parkinson's disease rating scale (MDS-UPDRS) part three and the Hoehn & Yahr staging system.^17^ Depressive symptoms were assessed with the ‘Beck Depression Inventory’ (BDI),^18^ overall cognition with the MoCA and non-motor symptoms with MDS-UPDRS part one. More details are available in the published protocol.^14^

### Anxiety assessment

The Parkinson anxiety scale (PAS), a scale specifically developed to detect and measure anxiety in PD patients, was used to assess anxiety symptoms. It is insensitive to motor and depressive symptoms and has subsections for persistent anxiety (PAS-A), episodic/situational anxiety (PAS-B), and avoidance behavior (PAS-C).^19^ A PAS-total score was calculated by summing the three subscores. PD-patients were considered to have significant PD-related anxiety if they had a score above the cut-off in at least one of the three subparts of the scale: PAS-A > 9, PAS-B > 3, or PAS-C > 3. Three groups were made: 1) PD-patients with anxiety (Anx-PD), 2) PD-patients without anxiety (non-Anx-PD), 3) healthy controls (HC).

### Imaging acquisition

Participants were scanned on a 7T MRI scanner (Magnetom, Siemens, Erlangen, Germany) equipped with a Nova Medical 32-channel head coil. Dielectric pads were applied to enhance the signal in the temporal brain regions. Cardiac and respiratory physiological signals were measured synchronized with the scan start. For this study, a whole-brain MP2RAGE (Magnetization Prepared 2 Rapid Acquisition Gradient Echoes) acquisition was used with an acquisition time of 10:57 min, resulting in a T1-weighted image and a quantitative T1map. These technical settings were used: TE = 2.51 ms, TR = 5000 ms, TI = 900 ms and 2750 ms, flip angle = 5° and 3°, FoV = 208 mm, resolution (x-y-z) = 0.65 × 0.65 × 0.65 mm3, slices = 240, orientation = sagittal (more details in the published protocol).^14^

### Thalamus and thalamic subnuclei segmentation

**Figure 1.**
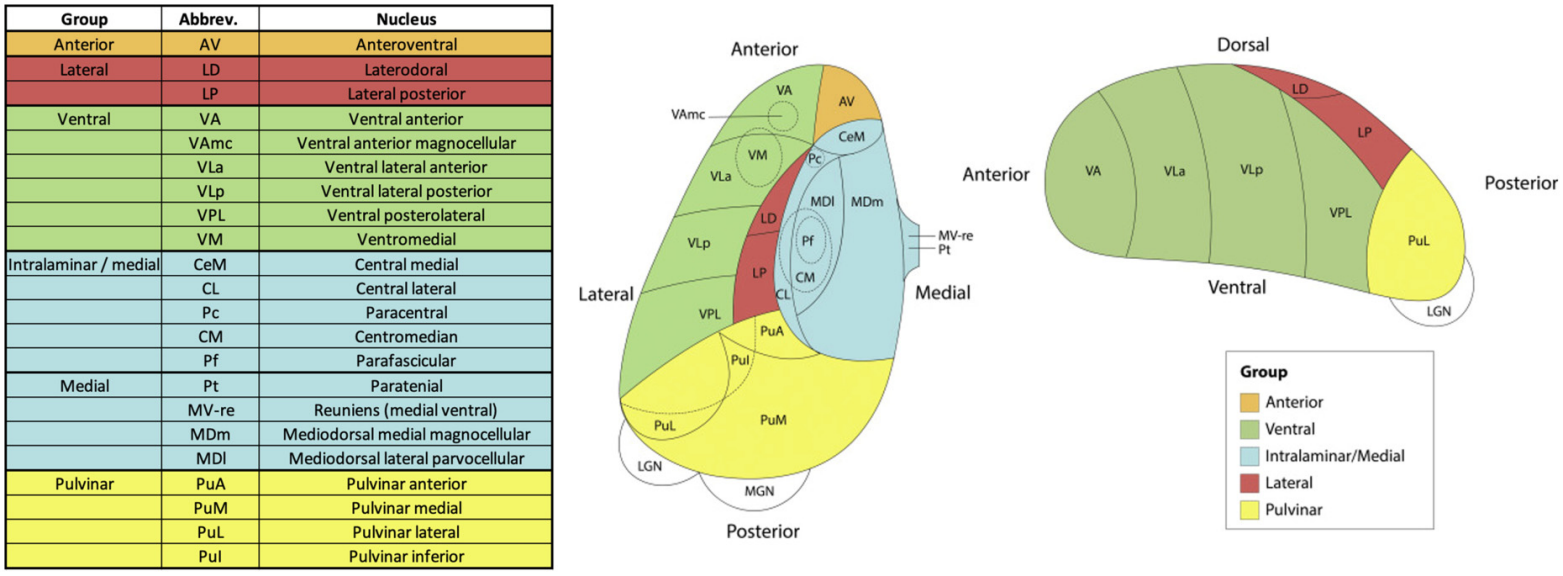
Schematic representation of the thalamic subnuclei and the five subregions. Figure adapted from Weeland et al. 2022.^22^

After dicom to nifti conversion, the MP2RAGE datasets were bias field corrected and aligned to the ACPC line using MIPAV software. The MP2RAGE structural brain MRI scans were processed using FreeSurfer (version 6.0.0) (https://surfer.nmr.mgh.harvard.edu) for segmentation of subcortical structures including the whole thalamus.^20^ A first visual quality control was performed for each segmentation. The pipeline of Iglesias et al.^12^ was used to segment the thalamus and its 25 nuclei (Figure 1). To make the analysis more powerful, we grouped these 25 nuclei into five different subregions per hemisphere: anterior, lateral, ventral, intralaminar/medial and pulvinar according to previous studies (Figure 1).^21,22^ A visual quality control was performed on all segmentations to check for errors. This was additionally verified by calculating a minimal or maximal threshold corresponding to the 2.2×interquartile range.^23^ No outlier was identified.

The volume of the whole thalamus and each of the thalamic subnuclei (in mm3) was extracted bilaterally using FSL. The volumes of each subregion were obtained by adding the corresponding thalamic subnuclei's volumes.

### Statistical analyses

The significance threshold was set at p-value <0.05 and corrected for multiple comparisons (FDR - False Discovery Rate) when necessary. The numerical variables were described as means and standard deviations, the ordinal variables as median and range and the categorical variables as frequencies and percentages. The normality of distribution was assessed using a Kolmogorov-Smirnov test.

### Descriptive analyses

Categorical data were compared with Chi2 tests and quantitative data with ANOVA tests, between the three groups, using SPSS, version 29 (SPSS, Chicago).

### Imaging analyses

First, the volumes of the whole thalamus and of the thalamic subregions were compared between the three groups using ANCOVA models. According to the literature, age and sex are associated with the volume of the thalamus,^22,24^ especially in PD patients.^25^ Moreover, according to our previous studies, anxiety symptoms are associated with depressive symptoms and sex.^5,26^ Finally, the thalamic volume had to be normalized by the total brain volume. Therefore, in this ANCOVA model, age, sex, depressive symptoms (BDI-score) and total brain volume were set as covariables.

Secondly, hierarchical multiple regression analyses were performed to examine the relationship between the PAS-total score and the volume of the subregions. Age, sex, depressive symptoms (BDI-score) and total brain volume were set as nuisance regressors in the first block (model 1) of all regression models, whereas PAS-total score (independent variable) was separately added to the second block of the model (model 2). The volume of the analyzed subregion was set as dependent variable. We ensured that all models met the assumptions for multiple regression analyses, including normality of the residuals, multicollinearity, and homoscedasticity. In case of significant differences, the same analyses were performed for each subnucleus of the corresponding subregion. The same statistical analyses were performed with the PAS sub scores A, B, and C.

### Reproducibility analyses

In order to verify the findings, the thalamic subnuclei were segmented a second time using a another automatic method, the THOMAS pipeline provided by Su et al.^27,28^ (more details in Supplemental Methods). The thalamic segmentation methods from Iglesias et al. (FreeSurfer) and from Su et al. (THOMAS) are both equally recommended according to a recent well documented study.^21^ Each scan and each nucleus was visually inspected and outliers were identified and excluded from the analyses (n = 3) using the same method as previously described (2.2× interquartile range). The same statistical methods were used.

## Results

### Population and descriptive analysis

Among the 151 participants included in TRACK-PD study, there were 105 PD patients, 45 healthy controls and one PD patient excluded because of MRI refusal. Among the 105 PD patients, 31 (29.5%) had clinically significant anxiety while 74 (70.5%) had not. The BDI total score was higher in the Anx-PD group than in the other groups as were the scores on the MDS-UPDRS 1.1 (cognitive disorders), 1.3 (depressive mood), 1.4 (anxiety state), and 1.5 (apathy) items. The results are detailed in Table 1.

**Table 1.** Descriptive analyses between Parkinson's disease patients with anxiety (Anx-PD), without anxiety (non-Anx-PD) and healthy controls (HC).

Variables	Anx-PD (n = 31)	Non-Anx-PD (n = 74)	HC (n = 45)	p-value
Demographic variables				
Sex (women)	10 (32.3%)	22 (29.7%)	14 (31.1%)	0.97
Age (y)	62.9 (± 9.19)	62.1 (± 8.13)	60.7 (± 8.07)	0.48
Dominant hand (right)	28 (90.3%)	62 (83.8%)	42 (93.3%)	0.27
Disease duration	1.64 (± 0.84)	1.61 (± 0.76)	NA	0.54
Side onset (right)	12 (46.2%)	39 (55.7%)	NA	0.40
LEDD	467.0 (± 261.53)	373.2 (± 209.88)	NA	0.094
Cognitive variable				
MoCA Total	27.7 (± 1.56)	28.0 (± 1.68)	27.7 (± 1.56)	0.57
Clinical variables				
MDS-UPDRS 1				
1–1 Cognitive disorders	1 (0–2)	0 (0–2)	NA	**0.02**
1–3 Depressive mood	0 (0–3)	0 (0–2)	NA	**0.008**
1–4 Anxious state	1 (0–2)	0 (0–2)	NA	**<0.0001**
1–5 Apathy	0 (0–2)	0 (0–1)	NA	**<0.001**
MDS-UPDRS part 3	15.8 (± 4.87)	20.5 (± 6.99)	NA	0.51
Hoehn & Yahr stage	2 (1–2)	2 (1–3)	NA	0.64
PAS total score	15.55 (± 4.04)	5.70 (± 3.40)	5.98 (± 4.35)	**<0.001**
PAS-A: persistent anxiety	8.00 (± 2.89)	3.86 (± 2.48)	3.71 (± 2.60)	**<0.001**
PAS-B: episodic anxiety	4.10 (±1.87)	0.96 (± 0.99)	1.33 (± 1.64)	**<0.001**
PAS-C: avoidance behavior	3.45 (± 1.93)	0.88 (±0.86)	0.93 (±1.27)	**<0.001**
Depression score (BDI)	11.9 (± 5.71)	6.0 (± 3.59)	3.51 (±3.60)	**< 0.001**

BDI: Beck depression inventory; LEDD: levodopa equivalent daily dose; MoCA: Montreal Cognitive Assessment; PAS: Parkinson anxiety scale.

Bold = significant difference (p-value < 0.05).

### Comparison of thalamic volume and thalamic subregions volumes

There were no between-group differences in the volume of the whole thalamus and the thalamic subregions (Table 2).

**Table 2.** Comparison of the volume of the thalamus and the thalamic subregions between Parkinson's disease patients with anxiety (Anx-PD), without anxiety (non-Anx-PD) and healthy controls (HC) adjusted by sex, age, total brain volume and depressive symptoms according to BDI score.

ROI	Anx-PD (n = 31)	non-Anx-PD (n = 74)	HC (n = 45)	F-score	FDR p-value
Whole Thalamus					
Right Thalamus (mm^3^)	5658.0 (±705.5)	5579.1 (±580.2)	5444.7 (±615.4)	2.70	0.20
Left Thalamus (mm^3^)	5548.5 (±750.8)	5563.5 (±559.0)	5367.2 (±648.5)	0.11	0.29
Thalamic subregions: right hemisphere					
Anterior region (mm^3^)	118.1 (±19.0)	115.7 (±21.3)	117.8 (±23.5)	1.03	0.36
Lateral region (mm^3^)	121.1 (±25.3)	118.1 (±20.4)	124.9 (±25.4)	2.5	0.20
Ventral region (mm^3^)	2512.9 (±365.3)	2520.9 (±302.4)	2455.7 (±310.9)	1.59	0.25
Intralaminar-medial region (mm^3^)	1323.7 (±178.3)	1311.6 (±152.8)	1268.5 (±133.0)	2.04	0.20
Pulvinar (mm^3^)	1552.3 (±193.3)	1512.9 (±171.9)	1477.9 (±181.4)	2.26	0.20
Thalamic subregions: left hemisphere					
Anterior region (mm^3^)	104.6 (±15.8)	101.0 (±21.7)	97.7 (±21.0)	1.13	0.39
Lateral region (mm^3^)	122.5 (±31.5)	123.7 (±29.5)	131.8 (±29.3)	0.75	0.47
Ventral region (mm^3^)	2403.8 (±357.9)	2420.5 (±266.5)	2316.4 (±291.9)	1.96	0.29
Intralaminar-medial region (mm^3^)	1275.2 (±188.5)	1274.2 (±144.9)	1212.9 (±140.8)	3.00	0.29
Pulvinar (mm^3^)	1642.4 (±231.6)	1644.1 (±185.7)	1608.3 (±239.5)	1.18	0.39

BDI: Beck Depression Inventory; FDR: false discovery rate.

### Multiple regression analyses

There was no significant association between the PAS-total score and the thalamus or thalamic subregions’ volume in the HC group nor in the non-Anx-PD groups or in all PD-patients (Anx-PD group and non-Anx-PD group).

In the Anx-PD group, there was a strong and significant negative association between the volume of the right intralaminar-medial subregion and the PAS-total score (R = 0.80, FDR p-value_model _< 0.001, ß_PAS _= −0,330, p-value_PAS _= 0.02) and between the left anterior subregion and the PAS-total score (R = 0.73, FDR p-value_model _= 0.002, ß_PAS _= −0.407, p-value_PAS _= 0.01) (Table 3A).

**Table 3.** Hierarchical multiple regression between the PAS-total score and (A) the volume of the thalamic subregions and (B) and the volume of the thalamic subnuclei involved in the intralaminar-medial subregion or in the anterior subregion in Parkinson's disease patients with anxiety adjusted by age, sex, total brain volume and BDI score.

ROI	F-score	FDR p-value_model_	R	Beta_PAS_	p-value_PAS_
A. Regression with thalamic subregions					
Right hemisphere					
Anterior region	1.91	0.13	0.53	−0.154	0.43
Lateral region	2.49	0.07	0.58	0.134	0.47
Ventral region	**6**.**30**	**0**.001*	0.75	−0.120	0.43
Intralaminar-medial region	**9**.**09**	**<0**.**001**	**0**.**80**	**−0**.**330**	**0**.**02**
Pulvinar	5.26	0.002^*#^	0.72	−0.124	0.44
Right Thalamus	**9**.**45**	**<0**.001*	0.81	−0.179	0.19
Left hemisphere					
Anterior region	**5**.**79**	**0**.**002**	**0**.**733**	**−0**.**407**	**0**.**01**
Lateral region	2.23	0.08	0.31	−0.026	0.89
Ventral region	**5**.**61**	**0**.002*	0.72	−0.087	0.58
Intralaminar-medial region	**8**.**53**	**<0**.001*	0.79	−0.151	0.28
Pulvinar	**4**.**40**	**0**.006*	0.68	−0.056	0.74
Left Thalamus	**7**.**92**	**<0**.001*	0.78	−0.106	0.46
B. Regression with thalamic subnuclei (only significant results)					
Right hemisphere					
MDm: Mediodorsal medial magnocellular nucleus	**7**.**78**	**<0**.**001**	**0**.**78**	**−0**.**398**	**0**.**01**
MDl: Mediodorsal lateral parvocellular nucleus	**4**.**52**	**0**.**006**	**0**.**69**	**−0**.**394**	**0**.**02**
Left hemisphere					
Antero-ventral nucleus	**5**.**79**	**0**.**002**	**0**.**733**	**−0**.**407**	**0**.**01**

BDI: Beck Depression Inventory; FDR: false discovery rate; PAS: Parkinson Anxiety Scale.

Bold = significant difference (p-value < 0.05); ^#^effect of the variable sex (and not PAS); *effect of the variable total brain volume (and not PAS).

The volume of the subnuclei involved in the intralaminar-medial subregion were analyzed post-hoc, i.e., the central medial, central lateral, paracentral, centro-median and parafascicular nuclei for the intralaminar subregion and the paratenial, medial ventral reuniens, mediodorsal magnocellular and mediodorsal parvocellular nuclei for the medial subregion (Figure 1).^20^

The anterior region corresponded to the anteroventral nucleus in the thalamic atlas.

**Figure 2.**
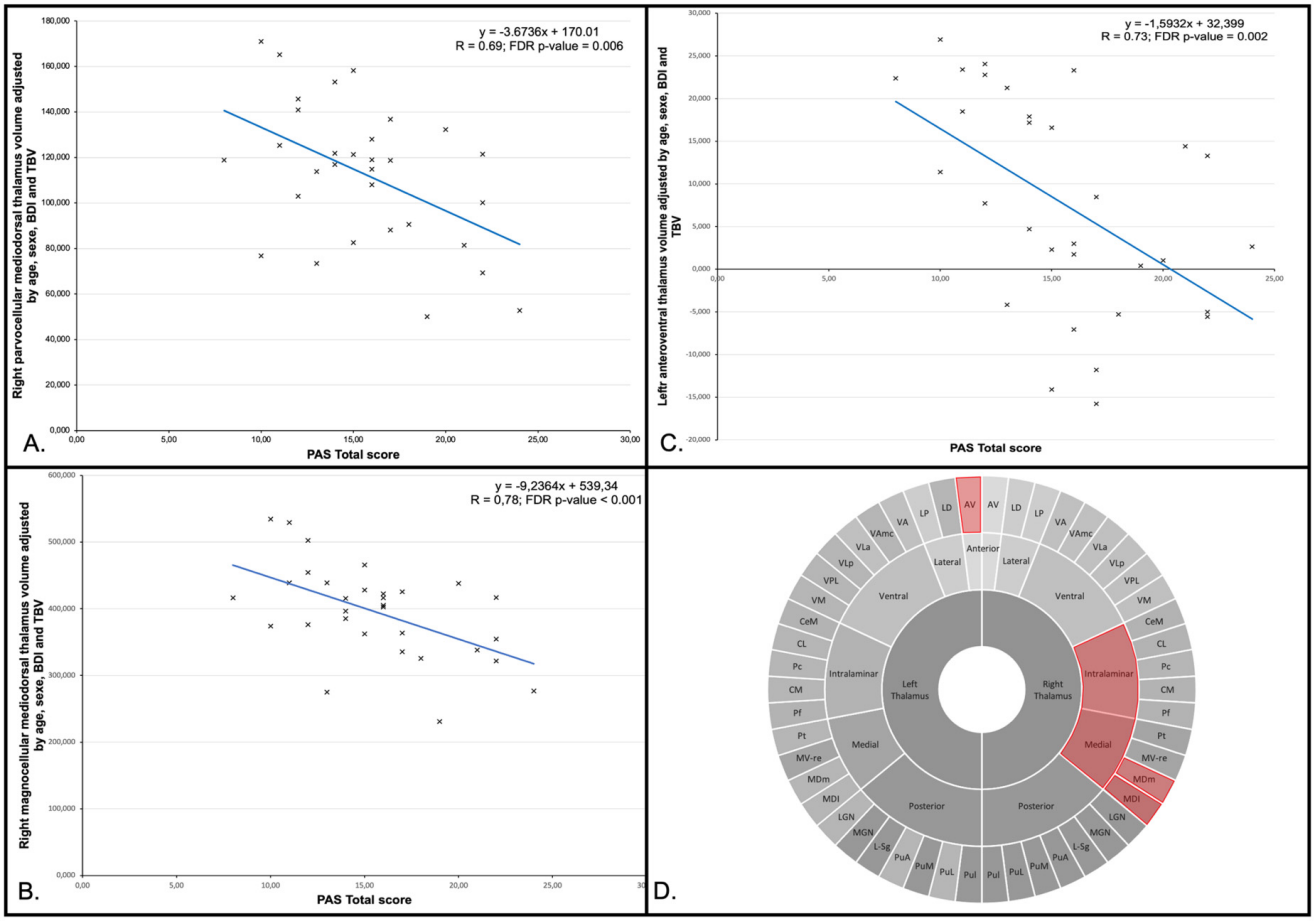
Scatterplot of the regression analyses between the right parvocellular mediodorsal thalamus (A), the right magnocellular mediodorsal thalamus (B) and the left anteroventral thalamus (C) volumes adjusted by age, sexe, Beck depressive inventory (BDI) and total brain volume (TBV) and the Parkinson anxiety scale (PAS) total score in Parkinson's disease (PD) patients with anxiety. Schematic illustration of reduced thalamic nuclei (in red) associated with PD-related (D) adapted from Chibaatar et al.^29^

In Anx-PD group, the volumes of the right MDm and left MDl had a strong negative association with the PAS-total score (R = 0.63, ß_PAS _= −0.546, p-value_model _= 0.007 and R = 0.60, ß_PAS _= −0.547, p-value_model _= 0.016, respectively) (Table 3B and Figure 2). There were no significant associations in the HC, non-Anx-PD groups.

AV: anteroventral; LD: laterodorsal; LP: lateral posterior; VA: ventral anterior; VAmc: ventral anterior magnocellular; VLa: ventral lateral anterior; VLp: ventral lateral posterior; VPL: ventral posterolateral; VM: ventromedial; CeM: central medial; CL: central lateral; Pc: paracentral; CM: centromedian; Pf: parafascicular; Pt: paratenial; MV-re: medial ventral reuniens; MDm: mediodorsal medial magnocellular; MDI: mediodorsal lateral parvocellular; LGN: lateral geniculate; MGN: medial geniculate; L-Sg: limitans suprageniculate; PuA: pulvinar anterior; PuM: pulvinar medial; PuL: pulvinar lateral; PuI: pulvinar inferior; FDR: False discovery rate.

There was no significant association between the PAS subscores and changes in thalamic volume in the total sample, nor in the PD subgroups with and without anxiety. In the anxious PD group, there were significant negative associations between the PAS-A subscore and volume of the right mediodorsal magnocellular thalamus (R = 0.79; p_model _< 0.001; p_PAS_A _= 0.02; β_PAS_A _= −0.35) and left anterior thalamus (R = 0.72; p_model _= 0.001; p_PAS_A _= 0.01; β_PAS_A _= −0.45), as well as between the PAS-C subscore and the volume of the right anterior thalamus (R = 0.62; p_model _= 0.03; p_PAS_C _= 0.04; β_PAS_C _= −0.36) (Supplemental Table 1).

### Reproducibility analyses

The analyses performed with the THOMAS pipeline provided similar results compared to our main analysis. There were no between-group differences. We found negative associations between the PAS-total score and anterior (R = 0.73; p-value = 0.001) and medial regions (R = 0.69; p-value = 0.005) of the thalamus. The results are described in detail in Supplemental Table 2.

## Discussion

This study aimed to identify anatomical differences in thalamic volume and its subnuclei between PD patients with anxiety and those without anxiety, using 7-Tesla MRI scanning. We found no differences in thalamic volume or its subregions between PD patients with or without anxiety, and healthy controls. However, in PD patients with anxiety, a higher severity of anxiety was associated with a smaller volume of the anterior thalamic subregion and the right mediodorsal thalamic subregion, more specifically the right mediodorsal magnocellular nucleus and the right mediodorsal parvocellular nucleus.

### Anxiety severity in PD is associated with a smaller anteroventral thalamus

**Figure 3.**
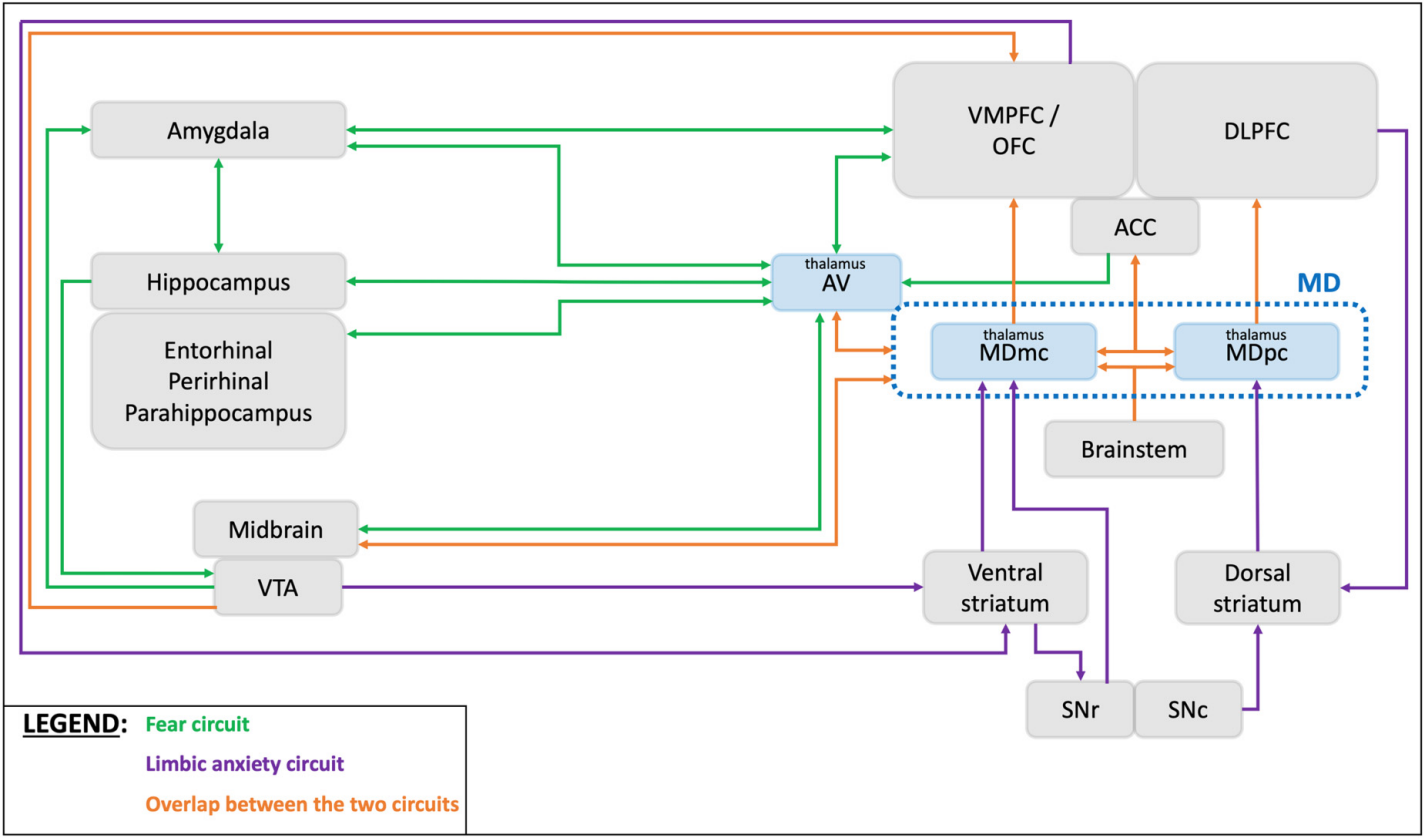
Schematic representation showing the structural connectivity of the anteroventral thalamus (AV), mediodorsal magnocellular thalamus (MDmc) and the mediodorsal parvocellular thalamus (MDpc), adapted from Pergola et al. and Nelson.^39,40^

ACC: anterior cingular cortex; DLPFC: dorsolateral prefrontal cortex; MD: mediodorsal thalamus; OFC: orbitofrontal cortex; SNc: substantia nigra pars compacta; SNr: substantia nigra pars reticulata; VMPFC: ventromedial prefrontal cortex; VTA: ventral tegmental area.

The anteroventral thalamus (AVT) is known to be part of an associative-cognitive grouping of nuclei with the laterodorsal, mediodorsal, reuniens and parateniens nuclei and involved in learning, memory, cognition and emotion regulation.^30^ It is a significant part of the limbic system. More specifically, the AVT is structurally connected to the nucleus accumbens, hypothalamus, hippocampus, amygdala, temporal cortex, orbitofrontal cortex, medial PFC and anterior cingulate cortex as well as the midbrain (Figure 3).^31^ Therefore, this nucleus can be considered to be part of the fear circuit. Structural alteration of anterior thalamus and its connections has been associated with anxiety-like behavior in mice,^32^ while a reduced volume of the anterior thalamus has already been linked with anxiety symptoms in humans.^30^ Reduced volume of the anterior thalamus was also found in other neuropsychiatric symptoms such as obsessive-compulsive disorder and depression.^22,29^ Interestingly, in a recent study, acute ischemic stroke involving the anterior thalamus but not the other thalamic regions would lead to long term anxiety symptoms.^33^ These different findings suggest that lesioning the anterior thalamus could lead to neuropsychiatric symptoms and especially anxiety in the general population. In PD patients, changes in the volume of thalamic nuclei have been correlated with the diagnosis and the progression of the disease or with symptoms such as freezing of gait, rapid eye movement sleep behavioral disorders (RBD) or cognitive disorders. However, the results of these studies were inconsistent since a smaller thalamic volume was correlated with disease progression, the presence of RBD and mild cognitive impairment^34–36^ while a bigger thalamic volume was reported in case of freezing of gait.^37^ It is also suggested that PD patients have a bigger volume of the AVT at the onset of the disease compared with healthy controls.^38^ Therefore, the thalamus, namely the AVT subnucleus, is involved in PD but its exact role remains uncertain. In our study, severity of anxiety symptoms in PD patients was associated with a smaller AVT volume. Since the AVT is part of the fear circuit, its structural alteration could play a role in the inappropriate fear processing in PD-related anxiety.

### Anxiety severity in PD is associated with a smaller mediodorsal thalamus

The mediodorsal thalamus (MDT) is the largest nuclear structure of the medial thalamus. It is classically described with several subdivisions such as the magnocellular MDT and the parvocellular MDT.^41^ The MDT is mainly structurally connected with the PFC, limbic structures, and basal ganglia. These connections differ between the magnocellular and the parvocellular MDT. The magnocellular MDT has connections with the ventromedial PFC, the orbitofrontal cortex and the anterior cingular cortex, and receives afferents from the amygdala, the parahippocampal cortex, the midbrain, the brainstem, the substantia nigra, the ventral striatum and the ventral pallidum. The parvocellular MDT relates to the dorsolateral PFC and the anterior cingular cortex and receives afferents from the brainstem, the substantia nigra, the dorsal caudate and the rostral pallidum (Figure 3).^39,41^ Recently, an “emotional pathway” was described in primates between the amygdala, the orbito-frontal cortex and the MDT that could refer to the fear circuit. Moreover, it has been shown that the MDT is involved in a cortico-striatal-thalamic loop involved in emotion regulation.^42,43^ Animal studies showed that lesioning the MDT in rats or in mice leads to structural and functional alterations in the PFC as well as in the amygdala and enhances anxiety-like behaviors suggesting that MDT alteration could lead to anxiety disorders.^44,45^ In humans, reduced volume of the MDT was associated with occurrence of anxiety symptoms^30^ and other neuropsychiatric disorders such as depression and obsessive-compulsive disorders.^22,29^ In PD patients, studies found that reduced volume of the MDT would be involved in cognitive disorders and behavioral symptoms such as hallucinations and depression.^46–48^ A recent study reported that, compared to healthy controls, the volume of the MDT was smaller in PD patients at the onset of the disease.^38^ Similar changes were described in Lewy bodies disease, suggesting a possible involvement of MDT atrophy in other synucleinopathies.^49^

In our study, the severity of anxiety in anxious PD patients was strongly associated with a smaller volume of the magnocellular MDT and the parvocellular MDT. These results are consistent with previous studies from our group. Indeed, we reported a reduced functional connectivity between the orbitofrontal cortex and the thalamus, increased functional connectivity between the amygdala and the thalamus and changes in structural connectivity between the striatum and the thalamus in PD-related anxiety.^6,11,26^ MDT structural alterations in PD could alter the connection between the thalamus and the PFC, the anterior cingular cortex and the orbitofrontal cortex leading to loss of function in the anxiety-related circuits. This could alter the cognitive control of emotion within the PFC and the limbic structures. Furthermore, it could inappropriately activate the emotional/fear processes, i.e., the fear circuit, leading to anxiety symptoms.

### Mediodorsal and anteroventral thalamus: Interfaces between the anxiety-related circuits

As mentioned before, the AVT is involved in the fear circuit while the MDT is part of both the fear and the limbic anxiety circuits. These nuclei are interconnected highlighting again that these two anxiety-related circuits overlap.^31^ A previous study suggested that the functional interaction between the MDT dopaminergic receptors and the PFC glutamatergic (NMDA) receptors may be partially involved in anxiogenic-like behaviors in rats.^50^ We mentioned before that MDT atrophy occurred in PD and in anxiety disorders while AVT atrophy was mainly found in anxiety disorders. We speculate that neuronal loss due to PD would alter the cortico-striatal circuits by inducing atrophy in their structures. It could lead to MDT structural alterations and then dysfunction of the fear circuit. This could promote AVT structural alterations and worsen the dysfunction in the fear circuit resulting in anxiety symptoms. Therefore, these shared structures in the pathophysiology of both PD and anxiety disorders may explain the high prevalence of PD-related anxiety (Figure 3). However, the involvement of other structures such as the striatum, the ventral tegmental area or the brainstem nuclei may also be essential and still need to be explored. Further studies are needed to confirm this hypothesis.

### Strength and limitations

Strengths of this study are the large sample of participants with both PD patients and healthy controls, all scanned with high-field 7-Tesla MRI. So far, no study has used high-field MRI to study anxiety in PD with this number of well-characterized patients. There are however also some limitations. We used a probabilistic and automated atlas to segment the thalamic subnuclei that is less accurate compared to manual segmentation. However, all segmentations were visually inspected, and we followed common methods validated in other studies.^22^ Moreover, we performed a reproductivity analysis to ensure the validity of our results and similar results were found with this second analysis. Although this second method is well validated with 7T MRI, it might lead to a loss of information, particularly for the MD, which is not segmented in magnocellular and parvocellular parts. As we hypothesized alterations in the volume of the MD, we choose to use the first atlas for our main analysis. Furthermore, in this study, we only focused on the volume of the thalamus and thalamic subnuclei/subregions. It would be interesting to include other methods such as functional MRI, diffusion tensor imaging or iron content, and other structures such as striatum, ventral tegmental area or brainstem nuclei. Finally, we only focused on cross-sectional data while TRACK-PD is a longitudinal cohort, with an ongoing follow-up. Further studies are needed to better understand the progression of anxiety symptoms with imaging features and to determine risk factor for triggering or worsening anxiety in PD.

## Conclusion

In PD patients with anxiety, a higher severity of anxiety was associated with a smaller volume of the left AVT and the right MD, specifically the right magnocellular MDT and the right parvocellular MD. Specific thalamic alteration in PD could promote the imbalance between the anxiety-related circuits potentially explaining the high prevalence of PD-related anxiety. Further studies are necessary to understand the role of other structures such as brainstem nuclei.

## Supplemental Material

sj-docx-1-pkn-10.1177_1877718X241308141 - Supplemental material for Reduced volume of the mediodorsal and anteroventral thalamus is associated with anxiety in Parkinson's disease: A cross-sectional 7-tesla MRI study Supplemental material, sj-docx-1-pkn-10.1177_1877718X241308141 for Reduced volume of the mediodorsal and anteroventral thalamus is associated with anxiety in Parkinson's disease: A cross-sectional 7-tesla MRI study by Guillaume Carey, Mark L Kuijf, Stijn Michielse, Amée F Wolters, Kathy Dujardin and Albert FG Leentjens in Journal of Parkinson's Disease
