# Supplementary material for: Reduced volume of the mediodorsal and anteroventral thalamus is associated with anxiety in Parkinson's disease: A cross-sectional 7-tesla MRI study
Source: J Parkinsons Dis. 2025 Feb 3;15(2):338–48. doi: 10.1177/1877718X241308141 (PMC13347415; doi:10.1177/1877718X241308141)
Supplement: sj-docx-1-pkn-10.1177_1877718X241308141 - Supplemental material for Reduced volume of the mediodorsal and anteroventral thalamus is associated with anxiety in Parkinson's disease: A cross-sectional 7-tesla MRI study [file sj-docx-1-pkn-10.1177_1877718X241308141.docx]

**Supplemental Material**

**Reduced volume of the mediodorsal and anteroventral thalamus is associated with anxiety in Parkinson’s disease: A cross-sectional 7-tesla MRI study**

**Supplemental Methods**

*Reproducibility analysis*

In order to verify the findings, the thalamic subnuclei were segmented a second time using a second automatic method, the pipeline provided by Su et al.^1^ Thalamus Optimized Multi-Atlas Segmentation (THOMAS) provides automatic segmentation of 12 thalamic nuclei using white-matter-nulled (WMn) Magnetization Prepared Rapid Gradient Echo (MPRAGE) sequence at 7T. In these reproducibility analyses, the new modified pipeline for accurate segmentation of T1w (SPGR, MPRAGE) data was used with a docker container HIPS-THOMAS. All the steps of the pipeline are detailed in the github website (https://github.com/thalamicseg/hipsthomasdocker/blob/main/README.md).

T1 3D MPRAGE scans were used for this segmentation. It provides 12 thalamic nuclei as shown in Supplemental Figure 1.


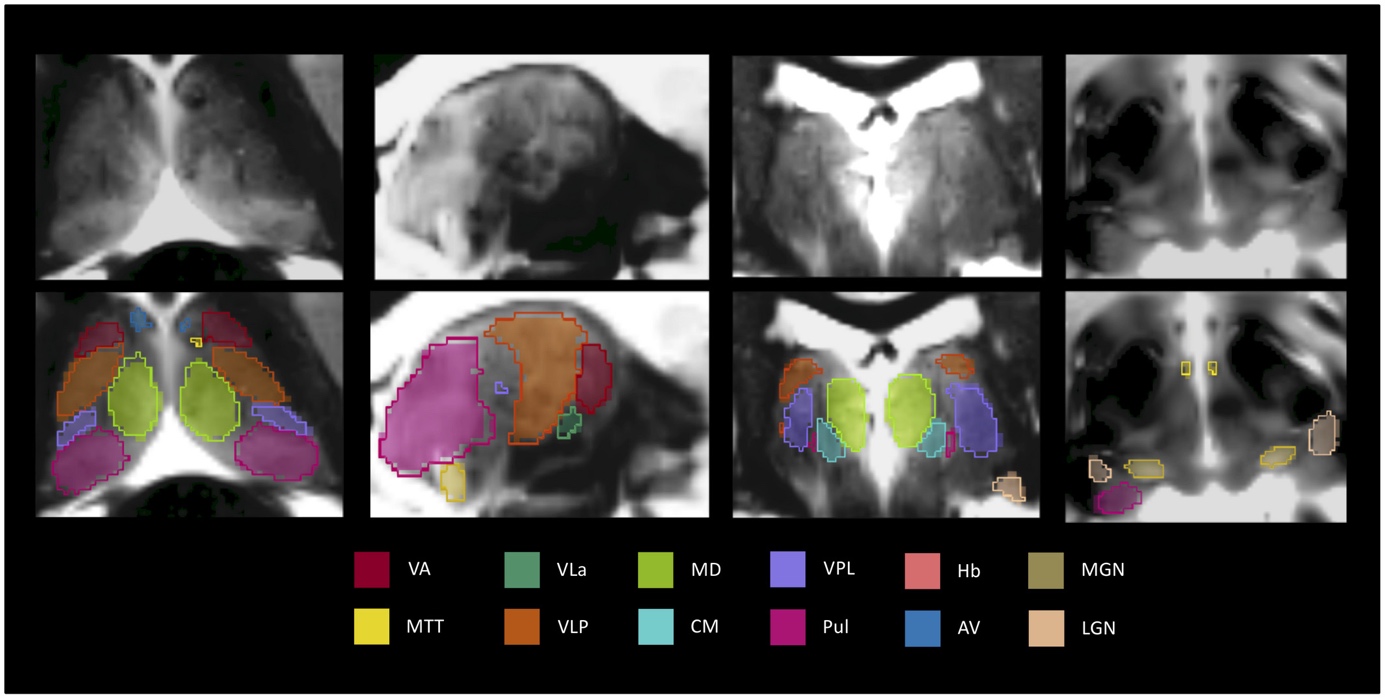


**Supplemental Figure 1.** A representative slice of automatic segmentation of different thalamic nuclei overlaid on a healthy subject (adapted from Datta et al.).^2^

AV: anteroventral nucleus; CM: centromedian nucleus; Hb: habenula; LGN: lateral geniculate nucleus; MD-Pf: mediodorsal–parafascicular complex; MGN: medial geniculate nucleus; MTT: mammillothalamic tract; Pul: Pulvinar ; VA: ventral anterior nucleus ; VLp: ventral lateral posterior nucleus; VLa: ventral lateral anterior nucleus; VPL: ventral posterolateral nucleus.

After the segmentation steps, a visual quality control was performed for all segmentations by GC to identify errors and failure of the process. Outliers were identify by calculating a minimal or maximal threshold corresponding to 2.2 x interquartile range. Three outliers were identified (1 HC and 2 nonAnxPD). They were excluded from the statistical analyses. The volume of each nuclei was provided by the THOMAS pipeline in mm^3^.

Group-comparison analyses and regressions analyses were done with the same methods than previous analyses.

**References**

1. Su JH, Thomas FT, Kasoff WS, et al. Fast, fully automated segmentation of thalamic nuclei from structural MRI. *Neuroimage* 2019: 194: 272-282.

2. Datta R, Bacchus MK, Kumar D, et al. Fast automatic segmentation of thalamic nuclei from MP2RAGE acquisition at 7 Tesla. *Magn Reson Med* 2021; 85: 2781–2790.

**Supplemental Results**

*Regression analyses with PAS subscores*

**Supplemental Table 1.** Hierarchical multiple regression between the PAS sub score and the volume of the thalamic subnuclei in Parkinson’s disease patients with anxiety adjusted by age, sex, total brain volume and BDI score.

| **ROI** | **F-score** | **p-value_model_** | **R** | **Beta _PAS_** | **p-value_PAS_** |  |
| --- | --- | --- | --- | --- | --- | --- |
| **Associations with PAS-A sub score (persistent anxiety)** | | | | | | |
| **Right hemisphere** | | | | | | |
| **Mediodorsal medial magnocellular thalamus** | 8.09 | <0.001 | 0.79 | -0.35 | 0.02 |  |
| **Left hemisphere** | | | | | | |
| **Anterior thalamus** | 5.51 | 0.001 | 0.72 | -0.45 | 0.01 |  |
| **Associations with PAS-B sub score (episodic anxiety)** | | | | | | |
| No significant association | | | | | | |
| **Associations with PAS-C sub score (avoidance behavior)** | | | | | | |
| **Right hemisphere** | | | | | | |
| **Anterior thalamus** | 3.12 | 0.03 | 0.62 | -0.36 | 0.04 |  |

BDI: Beck depression inventory; PAS: Parkinson anxiety scale.

Only significant results are provided

*Reproducibility analysis*

Regression analyses between PAS-total score and the volume of the thalamic subnuclei using THOMAS-pipeline.

**Supplemental Table 2.** Hierarchical multiple regression between the PAS-total score and the volume of the thalamic subnuclei using atlas in all participants, in Parkinson’s disease (PD) patients and in PD patients with anxiety adjusted by age, sex, total brain volume and BDI score.

| **ROI** | **R** | **p-value model** | **p-value PAS** | **Beta PAS** |
| --- | --- | --- | --- | --- |
| **All participants** | | | | |
| Left centro-median nucleus | 0.64 | <0.001 | 0.02 | -0.21 |
| Left ventral posterolateral nucleus | 0.49 | <0.001 | 0.08 | -0.18 |
| **PD patients (with or without anxiety)** | | | | |
| Left centro-median nucleus | 0.69 | <0.001 | 0.08 | -0.18 |
| **Anxious PD patients** | | | | |
| Right anteroventral nucleus | 0.73 | 0.001 | 0.06 | -0.30 |
| Right centro-median nucleus | 0.69 | 0.005 | 0.09 | -0.30 |
| Right mediodorsal – parafascicular nucleus | 0.69 | 0.004 | 0.09 | -0.21 |
| Left anteroventral nucleus | 0.66 | 0.004 | 0.08 | -0.29 |

BDI: Beck depression inventory; PAS: Parkinson anxiety scale.

Only significant results are provided.
